# A three-dimensional mathematical model for the signal propagation on a neuron's membrane

**DOI:** 10.3389/fncom.2015.00094

**Published:** 2015-07-17

**Authors:** Konstantinos Xylouris, Gabriel Wittum

**Affiliations:** Department of Simulation and Modeling, Faculty of Informatics, Goethe Center for Scientific Computing, Goethe University FrankfurtFrankfurt am Main, Germany

**Keywords:** models, theoretical, ephaptic coupling, dipole effect, detailed 3D-modeling, 3D-modeling, cable equation

## Abstract

In order to be able to examine the extracellular potential's influence on network activity and to better understand dipole properties of the extracellular potential, we present and analyze a three-dimensional formulation of the cable equation which facilitates numeric simulations. When the neuron's intra- and extracellular space is assumed to be purely resistive (i.e., no free charges), the balance law of electric fluxes leads to the Laplace equation for the distribution of the intra- and extracellular potential. Moreover, the flux across the neuron's membrane is continuous. This observation already delivers the three dimensional cable equation. The coupling of the intra- and extracellular potential over the membrane is not trivial. Here, we present a continuous extension of the extracellular potential to the intracellular space and combine the resulting equation with the intracellular problem. This approach makes the system numerically accessible. On the basis of the assumed pure resistive intra- and extracellular spaces, we conclude that a cell's out-flux balances out completely. As a consequence neurons do not own any current monopoles. We present a rigorous analysis with spherical harmonics for the extracellular potential by approximating the neuron's geometry to a sphere. Furthermore, we show with first numeric simulations on idealized circumstances that the extracellular potential can have a decisive effect on network activity through ephaptic interactions.

## Introduction

The membrane potential belongs to the most important quantities of a neuron. Its function of time and space describes neuronal activity. It is a voltage across the membrane defined by the difference between the intra- and extracellular potential.

Since the neuron is embedded in ionic milieus, potential gradients in the off-membrane spaces result in electric fluxes, which are conserved according to the first principles. This conservation law is the basis of the standard cable equation which describes the unfolding and propagation of an action potential (Rall, 1962, 1964; Scott, 1975) very efficiently. The standard cable equation maps a neuron to a tree of lines, each of which corresponds to a cylindric compartment with mean diameter. On these structures, it computes the evolution of the membrane potential according to its diffusion equation.

The resulting extracellular potentials can be theoretically computed with the line source method (Holt and Koch, 1999; Gold et al., 2006), once the transmembrane currents have been determined with the aid of the cable equation's solution.

These extracellular potentials in turn can be exploited to examine ephaptic feedbacks on other neurons (Holt and Koch, 1999). Indeed, the distribution of the extracellular potential can elicit transmembrane currents which may have decisive effects on the membrane potential of neighboring cells (Anastassiou et al., 2011; Buzsáki et al., 2012).

The goal of the current paper is to develop and implement an integrated three-dimensional model which synchronously captures both quantities, the membrane potential and the extracellular potential, during activity and which uses the neuron's geometry as it is instead of reducing it to cylindric compartments. The aim of such a model is to deepen the knowledge in signal processing and to carry out simulations on small networks of realistic neurons while having all these influences in action.

The work of Voßen et al. (2007) did a first step in the development of a generalized cable equation. It was built on the principle of the continuity of electric fluxes. Although the core model with the intra-, extracellular and membrane potential was correctly derived, the subsequent approach used to couple these unknowns and to solve them numerically resulted in major difficulties. The limit case to the standard cable equation evoked greater challenges and the simulations themselves were restricted to a very small time period of hundreds of micro seconds on a small part of a passive membrane.

The study of Xylouris et al. (2010) used a more direct approach for the coupling and generalized the existing model to active membranes. Nonetheless, although it was capable to reproduce action potentials, it still lacked in many characteristics of the signal processing, like the width of the propagating signal, the waveform of extracellular potential at activity and the computation on more complicated geometries. Indeed, computations on more complicated geometries diverged numerically. Furthermore, the membrane potential's defining equation in Xylouris et al. (2010) was the transmembrane current, which contains the time derivative of the membrane's capacitive property as only differential operator. The membrane potential's propagation was provided indirectly through the difference between the intra- and extracellular potential- thus making it actually hard to expect correct results for the spacial distribution. Moreover, as consequence, it produced vanishing transmembrane currents causing zero extracellular potentials and zero ephaptic interactions. This is why, the solving procedure with this direct coupling was of little use.

This paper introduces a completely new coupling of the unknowns. Therein, the defining equation for the membrane potential contains its own spacial differential operator. For the first time, we could carry out simulations on three-dimensionally resolved ideal neurons and on a small network of cells. This description, furthermore, allows for a proof that the extracellular potential distributes in the extracellular space like a current multipole. It will show that the only current monopole for a neuron exists at rest.

## Model

### Three-dimensional cable equation

Let Ω_in_ and Ω_out_ be domains in ℝ^3^ denoting the neuron's intra- and extracellular space, respectively, and ${\bar{\Omega }}_{\text{in}}\cap {\bar{\Omega }}_{\text{out}}=\Gamma$ the membrane, a two dimensional manifold embedded in ℝ^3^. Let $\Omega =\Omega_{\text{in}}\cup \Omega_{\text{out}}={\mathbb{R}}^{3}$ be the whole space. Let, furthermore, Φ_in_, Φ_out_, and *V*_*m*_ be the intra-, extracellular, and membrane potential, respectively. Φ will represent either Φ_in_ or Φ_out_.

The quantities σ_in_ and σ_out_ denote the intra- and extracellular conductivities, respectively. The normal *n*_in → out_ is the normal on the membrane Γ pointing from the intracellular space to the extracellular. We will need this quantities in order to define the fluxes. For the active transmembrane flux, we will just consider the Hodgkin–Huxley model for the sake of a simpler writing. There we have the sodium conductivity $g_{{\text{Na}}^{+}}$, the potassium conductivity $g_{{\text{K}}^{+}}$ and the leakage conductivity *g*_L_. The quantities $E_{{\text{Na}}^{+}}$, $E_{{\text{K}}^{+}}$, and *E*_L_ denote the reversal potentials of the indexed ions. The gating parameters *n*, *m*, *h* obey ordinary differential equations (Hodgkin and Huxley, 1952) and calibrate how much of the maximal possible ionic flux passes through the channel.

Considering the non-membrane conductivity (≈ 3$\frac{mS}{cm}$) (López-Aguado et al., 2001) and the dielectricity of water (≈1), Gary Holt demonstrated in his Ph.D. Thesis (Holt, 1997) that a possible non-membrane capacitor would discharge with a time constant of approximately 3 ns. Because this time scale is much faster than the one of the phenomena considered—the fast channel dynamics react on a μs-time scale—, it appears as good approximation to assume no capacitive properties for the non-membrane spaces (ρ = 0 in Ω_in_ and Ω_out_). Indeed, this is the basis of the derivation for the three dimensional cable equation. In addition, we will assume to have time invariant magnetic fields ($\frac{d\vec{B}}{dt}=0$). Then, Gauß's and Faraday's law satisfy root equations in the intra- and extracellular space, so that the conservative electric field can be expressed with the aid of a potential gradient. Combining this gradient with Gauß's law immediately leads to the Laplace equation for the potentials in the non-membrane spaces.

(1) $$\begin{array}{lll} \nabla \cdot \vec{E} & =\frac{\rho }{\epsilon \epsilon_{0}}\overset{\left(\rho =0\right)}{=}0,\; & \; \end{array}$$

(2) $$\begin{array}{lll} \nabla \times \vec{E} & =-\frac{d\vec{B}}{dt}\overset{!}{=}0,\; & \; \end{array}$$

(3) $$\begin{array}{lll} \Rightarrow \vec{E} & =-\nabla \Phi ,\; & \; \end{array}$$

(4) $$\begin{array}{lll} \Rightarrow -\Delta \Phi & =0.\; & \; \end{array}$$

The constants ϵ_0_ and ϵ are the dielectricities in vacuum and material, respectively.

Because of flux continuity, the flux across the membrane is continuous and must correspond to the flux emerging from the membrane dynamics [denoted with *j*_all_(*V*_*m*_)]. Hence,

(5) $$\begin{array}{ll} -\sigma_{\text{in}}\nabla \Phi_{\text{in}}\cdot n_{\text{in}\to \text{out}}=-\sigma_{\text{out}}\nabla \Phi_{\text{out}}\cdot n_{\text{in}\to \text{out}}=j_{\text{all}}\;\;\;\text{on}\;\Gamma . & \; \end{array}$$

**Figure 1:**
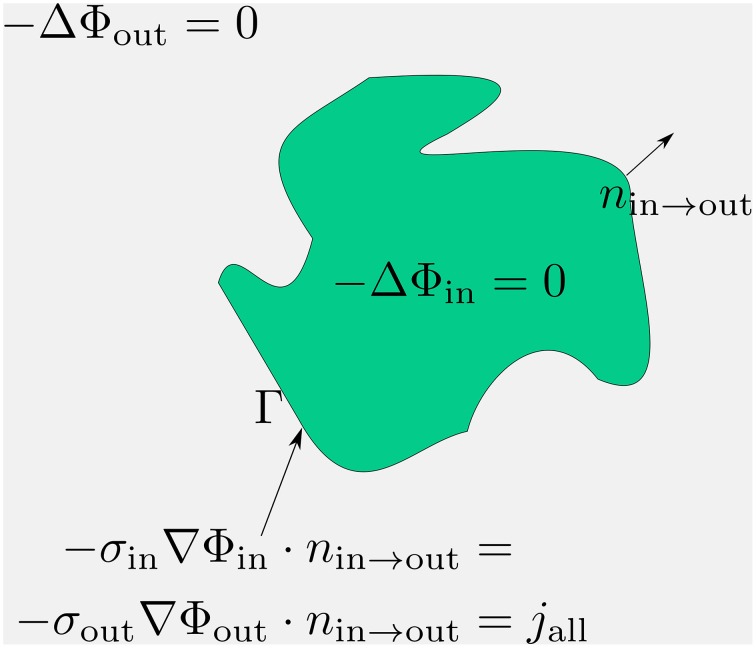
**Compact scheme of the three-dimensional cable equation**. Assuming pure resistivity for the non-membrane spaces, the respective potentials distribute according to the Laplace equation therein. These Laplace problems fulfill at the membrane an interface-condition which complies with the conservation of fluxes. The emerging flux from the potential equals the total transmembrane flux, denoted with *j*_all_. Within *j*_all_ all transmembrane currents are accumulated: capacitive, channel, any stimulation or synaptic currents.

With this boundary condition in mind, we arrive at the three-dimensional cable equation (Figure 1):

(6) $$\begin{array}{lllll} -\Delta \Phi_{\text{out}} & =0\; & \text{in}\;\Omega_{\text{out}}, & \; & \; \end{array}$$

(7) $$\begin{array}{lllll} -\Delta \Phi_{\text{in}} & =0\; & \text{in}\;\Omega_{\text{in}}, & \; & \; \end{array}$$

(8) $$\begin{array}{lllll} V_{m} & =\Phi_{\text{in}}-\Phi_{\text{out}}\; & \text{on}\;\;\Gamma . & \; & \; \end{array}$$

The flux *j*_all_ contains all fluxes passing the membrane. Considering just the Hodgkin–Huxley model and some additional stimulus, it looks like:

(9) $$\begin{array}{rcl} j_{\text{all}} & = & c_{m}\frac{dV_{m}}{dt}+m^{3}hg_{{\text{Na}}^{+}}\left(V_{m}-E_{{\text{Na}}^{+}}\right)+n^{4}g_{{\text{K}}^{+}}\left(V_{m}-E_{{\text{K}}^{+}}\right)\\ +g_{\text{L}}\left(V_{m}-E_{\text{L}}\right)+j_{\text{Stm}}. \end{array}$$

Since it is possible to have different dynamics on each region of the neuronal membrane, we furthermore introduce the following δ-functions

$$\begin{array}{lll} \delta_{\text{dend}}\left(x\right) & =\left\{\begin{array}{cc} 1 & \text{on the dendrite}\\ 0 & \text{else} \end{array}\right\},\; & \;\\ \delta_{\text{active}}\left(x\right) & =\left\{\begin{array}{cc} 1 & \text{on the soma or nodes of Ranvier}\\ 0 & \text{else} \end{array}\right\},\; & \;\\ \delta_{\text{syn}}\left(x\right) & =\left\{\begin{array}{cc} 1 & \text{on the postsynaptic density}\\ 0 & \text{else} \end{array}\right\},\; & \;\\ \delta_{\text{stim}}\left(x\right) & =\left\{\begin{array}{cc} 1 & \text{on the stimulation area}\\ 0 & \text{else} \end{array}\right\}.\; & \; \end{array}$$

With the help of these δ-functions, we can define a more refined transmembrane flux considering where it precisely occurs.

We define

(10) $$\begin{array}{rcll} j_{\text{HH}}\left(n,m,h,V_{m}\right) & = & m^{3}hg_{{\text{Na}}^{+}}\left(V_{m}-E_{{\text{Na}}^{+}}\right)+n^{4}g_{{\text{K}}^{+}}\left(V_{m}-E_{{\text{K}}^{+}}\right) & \\ +g_{\text{L}}\left(V_{m}-E_{\text{L}}\right). \end{array}$$

The synaptic activity is simply modeled with the aid of a modified Heaviside function *H*(*x*,*t*). This function should be one as soon as the membrane potential at the pre-synapse exceeds a certain value, say 2 mV, and it remains one for the time the synapse is active regardless of the presynaptic membrane potential. Additional activation at the pre-synapse should integrated by the synaptic function α(*V*_*m*_|_pre_,*t*)

(11) $$\begin{array}{ll} j_{\text{syn}}\left(V_{m}{|}_{\text{pre}},t\right)=H\left(V_{m}{|}_{\text{pre}},t\right)\cdot \alpha \left(V_{m}{|}_{\text{pre}},t\right), & \; \end{array}$$

where *V*_*m*_|_pre_ is the membrane potential at the presynaptic terminal.

Then the refined total transmembrane current has the form:

(12) $$\begin{array}{rcll} j_{\text{all}}\left(x,V_{m}\right) & = & c_{m}\frac{dV_{m}}{dt}+\delta_{\text{active}}\left(x\right)j_{\text{HH}}\left(n,m,h,V_{m}\right) & \\ +\delta_{\text{stim}}\left(x\right)j_{\text{Stm}}\left(t\right)+\delta_{\text{syn}}\left(x\right)j_{\text{syn}}\left(V_{m}{|}_{\text{pre}},t\right). \end{array}$$

### Numeric model

The three dimensional cable equation (Equations 6–8) is a non-symmetric system (Φ_in_ does not couple with Φ_out_ the same way as Φ_out_ with Φ_in_) of PDEs which couples two Laplace equations in the intra- and extracellular space with the transmembrane flux. This flux depends on the membrane potential. One difficulty in solving this system is the coupling of the membrane potential, which lives on a lower dimensional manifold, with the quantities, which live in full space. Since the discretization of this system is carried out with the help of integrals, the lower dimensional quantity cannot be measured the same way as the quantities in space (because the space integrals do not see it at all). In order to get rid of this particularity, we will extend the membrane potential, which is defined by the difference between the intra- and extracellular potential (*V*_*m*_ = Φ_in_ − Φ_out_) on the membrane, to the intracellular space. To that end, we extend the extracellular potential to the intracellular space and combine its extension with the intracellular potential equation. So, we arrive at a problem for the membrane potential in the intracellular space.

Because *V*_*m*_ = Φ_in_ − Φ_out_ on the membrane Γ, we will extend Φ_out_ to the intracellular space continuously so that the following identity holds. Let this extension be denoted with $\Phi_{\text{out}}^{IN}$:

(13) $$\begin{array}{lllll} V_{m} & =\Phi_{\text{in}}-\Phi_{\text{out}}=\Phi_{\text{in}}-\Phi_{\text{out}}^{IN}\; & \text{on}\;\;\Gamma , & \; & \; \end{array}$$

(14) $$\begin{array}{lllll} \Rightarrow \Phi_{\text{out}} & =\Phi_{\text{out}}^{IN}\; & \text{on}\;\;\Gamma . & \; & \; \end{array}$$

At this point we have some freedom to choose the right hand side of the extracellular potential extension equation. We choose it to be zero. Then it can be easily combined with the intracellular problem (Equation 7), which is a Lapalcian, too. We have

(15) $$\begin{array}{rcl} -\Delta \Phi_{\text{out}}^{IN} & = & 0\text{in}\;\Omega_{\text{in}}, \end{array}$$

(16) $$\begin{array}{rcll} \Phi_{\text{out}}^{IN} & = & \Phi_{\text{out}}\text{on}\;\Gamma , & \\ \Rightarrow -\Delta \left(\Phi_{\text{in}}-\Phi_{\text{out}}^{IN}\right)=-\Delta V_{m} & = & 0\text{in}\;\Omega_{\text{in}}, \end{array}$$

$$\begin{array}{rcll} -\sigma_{\text{in}}\nabla V_{m}\cdot n_{\text{in}\to \text{out}} & = & j_{\text{all}}\left(V_{m}\right) & \\ +\sigma_{\text{in}}\nabla \Phi_{\text{out}}^{IN}\cdot n_{\text{in}\to \text{out}} & \\ \text{on}\;\Gamma . & \end{array}$$

**Figure 2:**
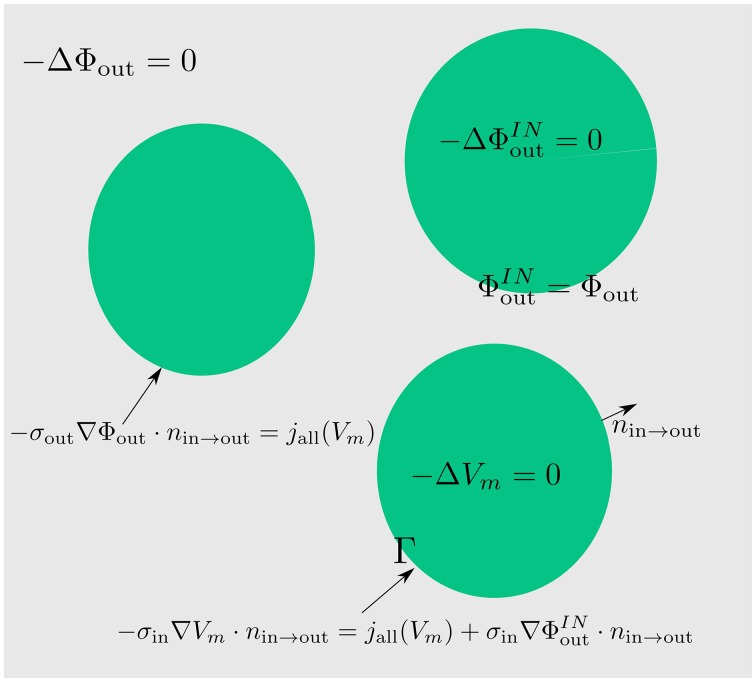
**By extending the extracellular potential to the intracellular space (green) continuously, an extension of the membrane potential into the intracellular space is established**. By means of this trick we obtain a coupling between the extracellular and the membrane potential which can be directly used for numerics and simulations.

Thus, instead of solving the system (Equations 6–8) we solve (Figure 2):

(17) $$\begin{array}{rcl} -\Delta \Phi_{\text{out}} & = & 0\text{in}\;\Omega_{\text{out}}, \end{array}$$

(18) $$\begin{array}{rcll} -\sigma_{\text{out}}\nabla \Phi_{\text{out}}\cdot n_{\text{in}\to \text{out}} & = & j_{\text{all}}\left(V_{m}\right)\text{on}\;\Gamma , & \\ -\Delta \Phi_{\text{out}}^{IN} & = & 0\text{in}\;\Omega_{\text{in}}, \end{array}$$

(19) $$\begin{array}{rcl} \Phi_{\text{out}}^{IN} & = & \Phi_{\text{out}}\text{on}\;\Gamma ,\\ -\Delta V_{m} & = & 0\text{in}\;\Omega_{\text{in}}, \end{array}$$

$$\begin{array}{rcl} -\sigma_{\text{in}}\nabla V_{m}\cdot n_{\text{in}\to \text{out}} & = & j_{\text{all}}\left(V_{m}\right)\\ +\sigma_{\text{in}}\nabla \Phi_{\text{out}}^{IN}\cdot n_{\text{in}\to \text{out}}\\ \text{on}\;\Gamma . \end{array}$$

For referencing reasons, we will call the additional current, which is considered in the boundary condition of the membrane potential equation (Equation 19), as ephaptic current

(20) $$\begin{array}{ll} j_{\text{eph}}:=\sigma_{\text{in}}\nabla \Phi_{\text{out}}^{IN}\cdot n_{\text{in}\to \text{out}}. & \; \end{array}$$

### Numeric discretization and procedures

In space, we discretize this system (Equations 17–19) with the finite volume method (Versteeg and Malalasekera, 2007). This method guarantees the local conservation of fluxes. This is necessary, because the model has been derived on this principle. Furthermore, important characteristics of the solution, as we will see in the following section depend on this conservation. In time, an implicit method is used while the non-linearity is resolved with the Newton method.

Similarly to the finite element method, we discretize the domain Ω with volume elements, for example tetrahedrals, whose edge points and edges form the grid Ω_*h*_, and we approximate the unknown functions (in our case *V*_*m*_,Φ_out_, and $\Phi_{\text{out}}^{IN}$) with a linear combination of shape functions. Our shape functions *b*_*j*_(*x*) have the property to be continuous and linear on each elements (*j* = 0,…,*#Ω*_*h*_ = *N*). They are as many as our grid points (*#Ω*_*h*_ = *N*) and are uniquely determined by the following defining conditions

(21) $$\begin{array}{lll} b_{j}\left(x_{k}\right) & =\delta_{jk}\;\;\;x_{j}\in \Omega_{h}\; & \; \end{array}$$

(22) $$\begin{array}{lll} b_{j}\left(x\right) & \;\;\text{is continuous and linear on each element } & \; \end{array}$$

We represent our unknown functions with these

(23) $$\begin{array}{lll} V_{m}\left(x,t\right) & =\overset{N}{\underset{j=0}{\sum }}v_{m_{j}}^{t}b_{j}\left(x\right),\; & \; \end{array}$$

(24) $$\begin{array}{lll} \Phi_{\text{out}}\left(x,t\right) & =\overset{N}{\underset{j=0}{\sum }}\phi_{{\text{out}}_{j}}^{t}b_{j}\left(x\right),\; & \; \end{array}$$

(25) $$\begin{array}{lll} \Phi_{\text{out}}^{IN}\left(x,t\right) & =\overset{N}{\underset{j=0}{\sum }}\phi_{{\text{out}}_{j}}^{IN,t}b_{j}\left(x\right).\; & \; \end{array}$$

Purpose of the discretization schema is to establish linear systems out of the differential Equations (17–19) which uniquely determine the unknowns coefficients $v_{m_{j}}^{t},\phi_{{\text{out}}_{j}}^{t},\phi_{{\text{out}}_{j}}^{IN,t}$ of these linear combinations. The upper index *t* should indicate that these coefficients are time dependent.

For the finite volume method, we need to construct a so called dual grid, which arises from the domain discretization and which is used in order to discretize the differential space operators. We call the elements of the dual grid control volumes. The volume elements of the dual grid are defined by the edge points which correspond to the barycenters of the initial tetrahedrals and the barycenters of its sides and edges. By this construction, we create as many control volumes as we have nodes in the grid Ω_*h*_. Let *B*_*k*_ be the control volume of the *k*-th grid node. We integrate the differential equations over this control volume and apply Gauß' integral theorem:

(26) $$\begin{array}{lll} -\Delta \Phi_{\text{out}}\left(x,t\right) & =0\; & \; \end{array}$$

(27) $$\begin{array}{lll} \int_{B_{k}}-\Delta \overset{N}{\underset{j=0}{\sum }}\phi_{{\text{out}}_{j}}^{t}b_{j}\left(x\right)dx & =-\int_{B_{k}}\overset{N}{\underset{j=0}{\sum }}\phi_{{\text{out}}_{j}}^{t}\Delta b_{j}\left(x\right)dx\; & \; \end{array}$$

$$\begin{array}{ll} =\int_{\partial B_{k}}\overset{N}{\underset{j=0}{\sum }}\phi_{{\text{out}}_{j}}^{t}\nabla b_{j}\left(x\right)\cdot \vec{n}\left(x\right)dS\left(x\right)\; & \;\\ =\overset{N}{\underset{j=0}{\sum }}\phi_{{\text{out}}_{j}}^{t}\int_{\partial B_{k}}\nabla b_{j}\left(x\right)\cdot \vec{n}\left(x\right)dS\left(x\right)\; & \;\\ =\overset{N}{\underset{j=0}{\sum }}\phi_{{\text{out}}_{j}}^{t}a_{kj}.\; & \; \end{array}$$

Because ∂*B*_*k*_ is a polyhedron and *b*_*j*_(*x*) is analytically known, the integrals $\int_{\partial B_{k}}\nabla b_{j}\left(x\right)\cdot \vec{n}\left(x\right)dS\left(x\right)=a_{kj}$ can be analytically computed. Furthermore, on the membrane these integrals equal to the transmembrane flux (Equation 12) which in general can also depend on other unknowns, like the gating variables or the membrane potential. Furthermore, this is the term which includes the time operator $\frac{d}{dt}$. We discretize our equation fully implicit and because this flux is not linear, we apply Newton's method to solve the emerging equations for each time step. Therein, the Jacobian of the system needs to be inverted, which we accomplish with high efficient iterative solvers. More precisely, we use a parallel ILU-preconditioned BiCGstab method (Barrett et al., 1987). All of this has been implemented with the use of the C++-library ug4 (Vogel et al., 2012), providing flexible numerical tools for these purposes.

## Results

The intracellular problem (Equation 7) is a Laplace problem with a Neumann boundary. We referred this to the approximation of purely resistive non-membrane spaces (i.e., the intra- and extracellular space do not contain any free charges). Thus, the driving force of the intracellular potential is given by its Neumann-flux on the boundary (i.e., the membrane). Now, integrating the Laplace equation over the whole neuron and applying Gauß's theorem yields an important constrain for the transmembrane currents: The fluxes are balanced out over the whole membrane at each point of time!

(28) $$\begin{array}{lll} -\Delta \Phi_{\text{in}} & =0\; & \; \end{array}$$

(29) $$\begin{array}{lll} \Rightarrow \int_{\Omega_{\text{in}}}-\Delta \Phi_{\text{in}}dx & =\int_{\Gamma }-\sigma_{\text{in}}\nabla \Phi_{\text{in}}\cdot n_{\text{in}\to \text{out}}dS\left(x\right)\; & \;\\ =\int_{\Gamma }j_{\text{all}}\left(V_{m}\right)dS\left(x\right)\overset{!}{=}0\; & \; \end{array}$$

There are at least two important implications of this situation. First, an influx at some point of the membrane, necessarily leads to an out-flux at some other point of the membrane with the same total amount of current. Moreover, this must happen simultaneously, since otherwise the condition is violated.

Second, the extracellular potential distributes like a multipole in the extracellular space.

### Dipole-like distribution of the extracellular potential for a idealized sphere neuron

Regardless of the neuron's shape, the extracellular potential equation (Equation 17) demonstrates that its only source is the transmembrane flux as expressed through its boundary condition. A current monopole of the extracellular potential would be defined by the overall transmembrane flux. Yet, this flux is always zero as shown before (Equation 29). Thus, there is no monopole component and the extracellular potential distributes in space like a current multipole. To get some quantitative idea of its distribution, we approximate the neuron's geometry to a sphere. Then, we are able to express the extracellular potential with a generalized Fourier series of spherical harmonics.

Let Ω_in_ = *B*_*R*_ be a sphere with radius *R* and Γ = ∂*B*_*R*_ its boundary. The spherical harmonics $Y_{l}^{m}\left(\theta ,\phi \right)$ satisfy the Laplace problem on this geometry:

(30) $$\begin{array}{lll} -\Delta Y_{l}^{m} & =0\; & \; \end{array}$$

(31) $$\begin{array}{lll} \Phi_{\text{out}}\left(r,\theta ,\phi \right) & =\sum_{l\geq 0}\overset{l}{\underset{m\geq -l}{\sum }}\left(b_{lm}r^{-\left(l+1\right)}\right)Y_{l}^{m}\left(\theta ,\phi \right)\; & \; \end{array}$$

(32) $$\begin{array}{lll} \Rightarrow -\Delta \Phi_{\text{out}} & =0.\; & \; \end{array}$$

The solution Φ_out_ is concretized by the coefficients *b*_*lm*_. These are determined by the transmembrane flux *j*_all_(*V*_*m*_):

(33) $$\begin{array}{ll} \frac{\partial \Phi_{\text{out}}}{\partial r}{|}_{r=R}=\sum_{l\geq 0}\overset{l}{\underset{m\geq -l}{\sum }}-\left(l+1\right)\frac{1}{R^{l+2}}b_{lm}Y_{l}^{m}\left(\theta ,\phi \right)=j_{\text{all}}\left(V_{m}\right) & \; \end{array}$$

(34) $$\begin{array}{ll} \Rightarrow b_{kn}=-\frac{R^{l+2}}{l+1}\int_{0}^{\pi }\int_{0}^{2\pi }sin\left(\theta \right)j_{\text{all}}\left(V_{m}\right)Y_{k}^{n}\left(\theta ,\phi \right)d\theta d\phi . & \; \end{array}$$

Especially, we obtain for the first coefficient *b*_00_ which corresponds to the potential of a monopole:

(35) $$\begin{array}{lll} b_{00} & =-\frac{R^{l+2}}{l+1}\int_{0}^{\pi }\int_{0}^{2\pi }sin\left(\theta \right)j_{\text{all}}\left(V_{m}\right)\frac{1}{\sqrt{4\pi }}d\theta d\phi \; & \;\\ =-\frac{R^{l+2}}{\left(l+1\right)\sqrt{4\pi }}\int_{\Gamma }j_{\text{all}}\left(V_{m}\right)dS\left(x\right)=0.\; & \; \end{array}$$

Thus, the solution of the extracellular potential does not contain any monopole-part and behaves like a multipole falling in space with higher powers of the distance.

### Numerical error analysis and verification by a comperison with NEURON

NEURON (Hines and Carnevale, 1997) is a highly sophisticated simulation environment for modeling a wide range of neuronal networks with the aid of the standard cable equation. Since the current three-dimensional model generalizes the one dimensional cable equation and since there are no non-trivial analytic solutions of an active neuron for our equations, we want to use this software environment in order verify both our model and our implementation. Our results should be very similar with these of NEURON for comparable computational domains. In order to keep the three-dimensional computation fast and in order to be able to create suitable three-dimensional computational domains, we carry out this comparison on a very long cylinder *l* = 9.9 mm with small diameter *d* = 200 μm in relation to its length ($\frac{d}{l}\approx 2\cdot 10^{-4}$). Such cases approximately comply with the assumption of the one-dimensional model ( of infinite cylinders). No significant differences in the rise and propagation of an arising action should be visible.

**Figure 3:**
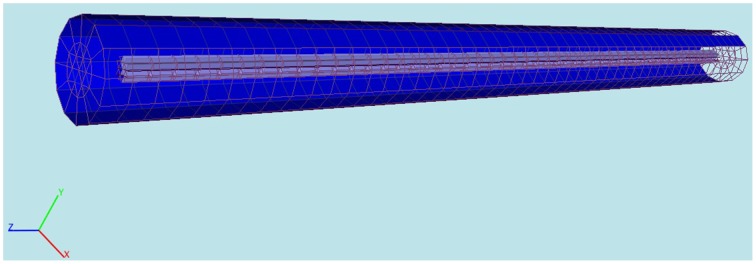
**Computational domain constructed with proMesh (Reiter, 2014) for the comparison of the 3D-model's results with NEURON**. The cylinder represents a soma having a length of 9.8 mm and a diameter of 200 μm. The purple is the intracellular and the blue the extracellular space. The domain is discretized with tetrahedrals.

We use proMesh (Reiter, 2014) to construct the three dimensional cylindric soma with a length 9.8 mm and a diameter 200 μm (Figure 3).

This test domain we now use in order to first verify the the correct implementation of our discretization schema and second in order to see that we indeed obtain almost identical solutions in comparison with those produced by NEURON.

First is obtained, if the computed solution converges as the computational grid fineness is increased. In order to assess the second point, we have to compare the one dimensional solution of NEURON with the three-dimensional solution of our model. By construction of the one dimensional cable equation, each quantity, although computed on every point of a line, actually represents a volumetric quantity. Thus, the one-dimensional model assumes for all quantities to be radial symmetric and iso-potential on cross-sections of a three-dimensional cylinder. Considering this particularity, we can blow up the solution of NEURON to a three-dimensional solution and compare it with the solution of our model or we compare NEURON's solution with our solution recorded on the cylinder axis. For the sake of simplicity, we use the second way considering that its difference with the volumetric comparison is just the factor of the cross-section area.

Because for three dimensional numeric computations, domains have to be discretized, even simple cylinders never correspond to ideal cylinders, which, however, are the basis of the one-dimensional model. Thus, we will always expect small quantitative differences in such a comparison and, therefore, we are already satisfied to evaluate the differences with NEURON with the aid of an Euclidean integral norm

(36) $$\begin{array}{ll} ||f|{|}_{L^{2}\left(\left[a,b\right]\right)}=\sqrt{\int_{a}^{b}|f{|}^{2}dx}, & \; \end{array}$$

where the interval [*a*,*b*] corresponds to the time interval of the simulation. Furthermore, in order to get this measure dimensionless, we will consider the relative error between the solution of neuron *V*_*m*_NEURON__ and the solution computed at refinement level *x*, denoted with *V*_*m*_Level *x*__, over the interval [0,*T*]

(37) $$\begin{array}{ll} \frac{||V_{m_{\text{NEURON}}}-V_{m_{\text{Level}\;x}}|{|}_{L^{2}\left(\left[0,T\right]\right)}}{||V_{m_{\text{Level}\;x}}|{|}_{L^{2}\left(\left[0,T\right]\right)}}. & \; \end{array}$$

Yet, qualitative measures like propagation speed and signal width should be identical.

**Figure 4:**
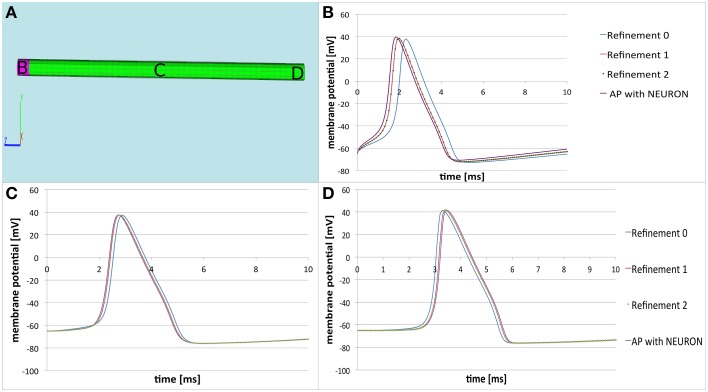
**Comparison of the three-dimensional model with NEURON**. **(A)** Computational domain with the marked areas **(B–D)** where the membrane potential is recorded. **(B–D)** Time courses of the membrane potential at the corresponding areas. The solution of the three dimensional model at refinement level 0 is the blue line. After two refinements the solution converges—the red line representing the solution at refinement level 1 coincides with the solution represented at refinement level 2 (dotted green line). This implies the correct implementation of the applied finite volume discretization schema. We see that the solution produced by the three-dimensional model (dotted green line) is almost the same as the solution produced by NEURON (purple line). The small differences are due to the nature of the three-dimensional modeling procedure (see text).

Concerning the numeric convergence at grid refinement, we computed the solution on our cylinder, composed by a tetrahedral grid, at two levels of refinement and observed the desired convergence (Figure 4). This behavior should serve as benchmark for the right implementation of the finite volume discretization schema.

The solution between the standard cable equation and the three dimensional model are qualitatively undistinguishable (Figure 3). The small numerical differences (Table 1) are due to the aforementioned reasons: the cylinder in the computation is a disretization of an ideal one, the cylinder's length is finite (the standard cable equation assumes infinite cylinders). Moreover, since the three-dimensional model additionally considers the coupling of the extracellular potential on the membrane, so that there are always to be expected some subtile differences in the solutions, which are reflected in Table 1.

**Table 1:** **Relative error of the computed solution in comparison with NEURON**.

**Solution on refinement level *x***	**$\frac{||V_{m_{\text{NEURON}}}-V_{m_{\text{Level}\;x}}|{|}_{L^{2}\left(\left[0,T\right]\right)}}{||V_{m_{\text{Level}\;x}}|{|}_{L^{2}\left(\left[0,T\right]\right)}}$**
*V_m_Level 0__*	0.2002
*V_m_Level 1__*	0.1174
*V_m_Level 2__*	0.1174

*The relative error between the solution computed with NEURON V_m_NEURON__ and the solution computed on refinement level x, denoted with V_m_Level x__ is very small. This implies that qualitative characteristics like propagation speed, signal width as well are very similar. The small differences measured here can be explained with the nature of the three-dimensional model which automatically considers the extracellular potential in the signal processing and which works with discretized and finite domains (in this case: cylinders are supposed to be ideal and infinite for the standard cable equation)*.

However as regards the emerging of the action potential (Table 1, Figure 4), the propagation speed of $5\frac{m}{s}$, and the signal width (Table 1, Figure 4) we receive identical results.

### Simulation on a small network of four idealized neurons

**Figure 5:**
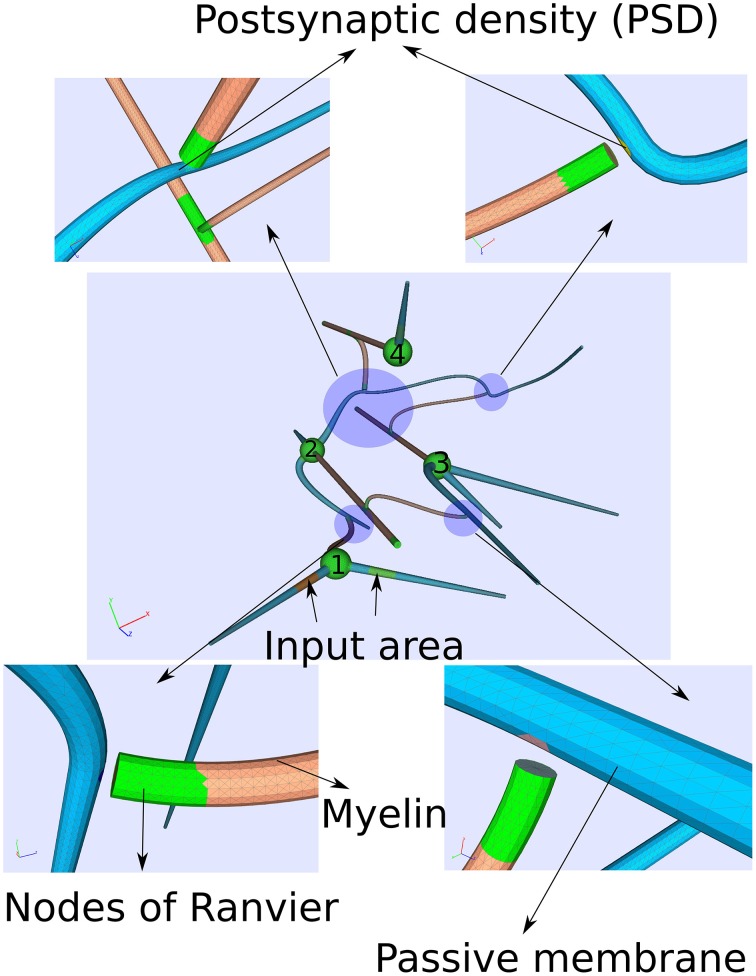
**Computational domain of four idealized neurons consisting of a soma, an axon, dendrites and chemical synapses**. Computationally, the synapses are modeled by a postsynaptic current at the postsynaptic site as soon the membrane potential exceeds some threshold indicating an action potential at the pre-synapse. Due to the complexity of the computation, a small time period of around 14 ms is covered.

With a computationally quite demanding simulation, we also solve the Equations (17–19) on a more complicated geometry representing four idealized neurons with chemical synapses (Figure 5).

The simulation is demanding, because we have a non-linear time-dependent domain problem in three dimensions. It means we solve several a huge linear systems in each time step within Newton's method. Thereby, the time step to be chosen is constrained by the fast dynamics of the active membrane's gating variables, which in our case is chosen with 10 μs, while we aim to simulate the time period of 14 ms. This means we need to compute the solution for 1400 time steps, which is time demanding despite parallel procedures due to the geometry's complexity.

We constructed the computational domain given by a small network of four neurons with the help of an algorithm developed in Niklas Antes' master thesis (Antes, 2009). Each cell consists of a myelinated axon (diameter *d* ≈ 5μm), a soma (*d* ≈ 20μm) and dendrites (*d* ≈ 10μm). The cells are several hundred micrometer separated among each other.

As regards the transmembrane current *j*_all_(*x*,*V*_*m*_) (Equation 12) for the different cell parts, we just considered passive properties on the dendrites while an active membrane reflecting Hodgkin–Huxley dynamics for the soma as well as for the nodes of Ranvier. On the myelinated sheaths, the transmembrane current *j*_all_(*x*,*V*_*m*_) is composed of the first term in Equation (12) only, the capacitive current. Furthermore, two of the cells (cell 1 and cell 4, see Figure 5) own external input areas by which the network can be stimulated.

Because we simulate the relatively small time period of 14 ms, we let the synapses work as pre-defined strong post-synaptic current pulses of some nA, which are triggered as soon as the membrane potential at the pre-synapse indicates that an action potential has arrived. This is assumed to happen when the membrane potential at the pre-synapse exceeds the value of 5 mV.

For the sake of simplicity, we choose a constant intra- and extracellular conductivity $\sigma_{\text{in}}=2\frac{mS}{cm},\sigma_{\text{out}}=20\frac{mS}{cm}$.

**Figure 6:**
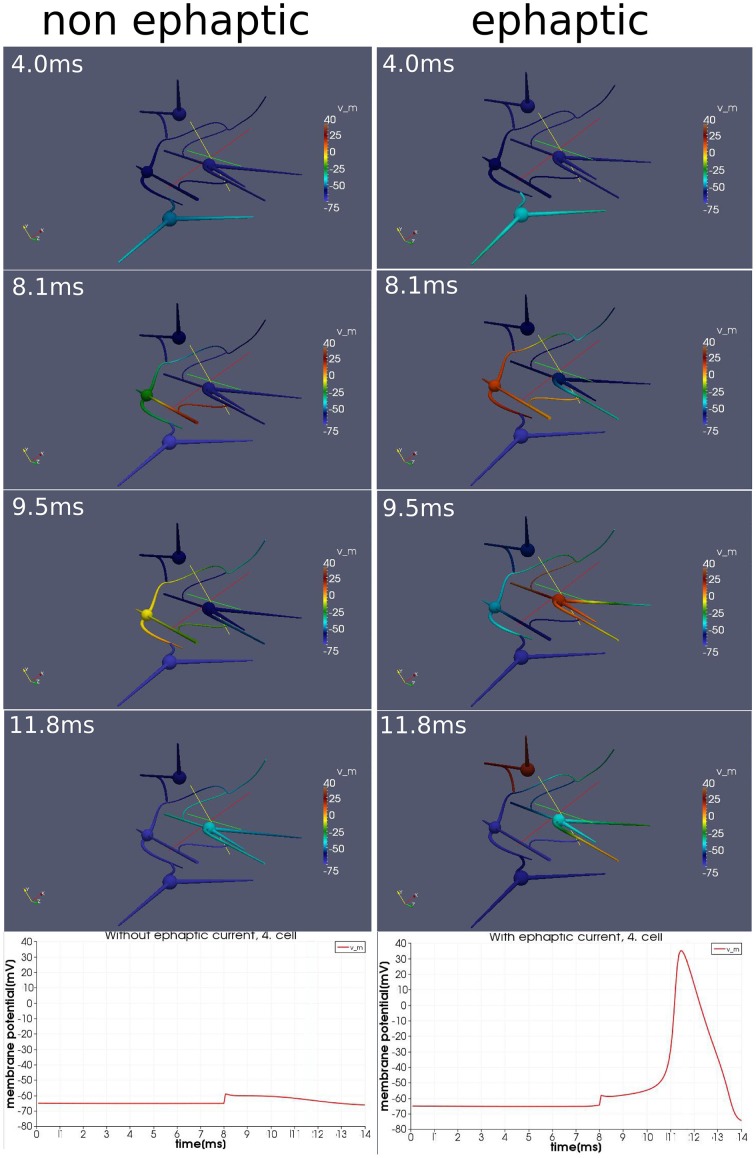
**Effect of ephaptic interactions**. Two simulations on an idealized small network of four cells and idealized paradigm. One simulation (left column), in which the ephaptic current (Equation 20) is neglected and one simulation (right column) in which it is included. Both simulations are carried out with the same stimulation paradigm. An initial signal spreads through the network. Additionally around the moment of 8 ms, the forth cell (the upper cell of this network) is activated slightly with a current pulse so that it depolarizes just below the threshold for and action potential. Although the effects of ephaptic interactions are very small, we see that they can determine whether a neuron activates in particular circumstances.

We activate the network by stimulating cell number one (see Figure 5) with approximately 30 pA at each of its input areas over the whole simulation period of 14 ms. At the moment of 8*ms*, we then stimulate cell number four with a current pulse of approximately 0.5 nA over 20μs. Although this stimulation of the fourth cell is not enough to generate an action potential alone, within the regime of this network and with the ephaptic current activated (Equation 20), an action potential arises (see Figure 6). This demonstrates that ephaptic interactions can have a decisive effect as to whether a neuron fires.

The model integrates the impact of the extracellular potential into the signal processing. Though its impact is rather small, it still can have a significant effect when combined with the right stimulation at the right time. Action potentials can arise, which otherwise would not show up (Figure 6).

## Discussion

The three-dimensional passive model of Voßen et al. (2007) has been extended to a model with active membrane dynamics and has been reformulated mathematically with the aid of an extension of the membrane potential into the intracellular space. This reformulation, for the first time, facilitated numeric simulations of neuronal activity on three-dimensionally resolved idealized neurons generalizing the one dimensional cable equation by fully incorporating the three-dimensional extension of the neurons' geometry and by automatically considering the extracellular potential's influence on the membrane. As shown, the latter influence -though it is quite small- in combination with additional stimulation at the right timing can lead to an action potential which otherwise would not have arisen.

For the sake of verifying the correct implementation of this model and because it should deliver similar results as the one-dimensional cable equation for the limit case of long and thin cylinders, we carried out a comparison with NEURON and obtained very good agreement between the two models.

Based on the assumption of charge-free non-membrane spaces -an assumption also used for the derivation of the standard cable equation-, we could provide strong theoretical evidence (to our knowledge for the first time) with the aid of the three-dimensional model that there aren't any current monopoles as the overall out-flux across the membrane balances out. A significant consequence of this behavior is that the leading term of the extracellular potential's multipole expansion vanishes so that it falls in space with higher powers of its distance to the transmembrane current source. In the work of Lindén et al. (2011), this very assumption has been applied for the extracellular potential in order to arrive at converging LFPs. The authors in Lindén et al. (2011) showed that a monopole behavior would lead to a diverging LFP.

We consider the ability to carry out realistic simulations with the cable equation on three-dimensionally resolved ideal neurons as important step and milestone on the way of refining and generalizing existing models for neuronal activity. This three dimensional model facilitates gaining a better understanding of all the processes involved in the signal processing, especially the influence of the extracellular potential activity on the membrane and the impact of the precise three-dimensional shape of the neuron's geometry. Concerning the ephaptic communication, it would be interesting to further investigate its influence on synchronous firing within networks. The latter point also seems to be very promising since lots of precise experimental geometric data are produced. Questions connecting function with geometry can be directly tackled with this model.

However, there is still a long way to go on this path, as the biggest challenge at the moment for our model is its computational demand. Further algorithmic and computational analysis needs to be invested in order to make applicable cutting edge solvers of linear systems arising from partial differential equations -like algebraic multi grid methods- on highly parallel machines, even on graphic card clusters. As next steps, we want to focus on these improvements.

On the other hand, the computational efficiency is a big advantage for standard one dimensional cable equation. Once we accomplished this efficiency for the three-dimensional model, there are still lots of interesting applications which we wish to address- especially concerning backward modeling with questions like which are the underlying network properties in order to reproduce a given a extracellular potential activity wave.

Furthermore, we see the need of a deeper theoretical analysis of this model with the purpose to provide a mathematical proof that it converges to the standard cable equation for the limit case of infinite cylinders and vanishing extracellular resistivity.

Our long-range purpose is to generalize this model with homogenization and multi-scale techniques so that to be able to simulate the activity of bigger clusters of neuronal networks while also considering the detail in processing on the small scale.

Realized steps on this path will be hopefully items of future publications.

### Conflict of interest statement

The authors declare that the research was conducted in the absence of any commercial or financial relationships that could be construed as a potential conflict of interest.

## References

[B1] Anastassiou C. A. Perin R. Markram H. Koch C. (2011). Ephaptic coupling of cortical neurons. Nat. Neurosci. 14, 217–223. 10.1038/nn.2727 21240273

[B2] Antes N. (2009). Ein Werkzeug zur Erzeugung von Oberächengeometrien von Neuronen. Master's Thesis, University of Heidelberg.

[B3] Barrett R. Berry M. Chan T. F. Demmel J. Donato J. Dongarra J. (1987). Templates for the Solution of Linear Systems: Building Blocks for Iterative Methods, Vol. 43. Philadelphia, PA: Society for Industrial and Applied Mathematics.

[B4] Buzsáki G. Anastassiou C. A. Koch C. (2012). The origin of extracellular fields and currents EEG, ECOG, LFP and spikes. Nat. Rev. Neurosci. 13, 407–420. 10.1038/nrn3241 22595786 PMC4907333

[B5] Gold C. Henze D. A. Koch C. Buzsáki G. (2006). On the origin of the extracellular action potential waveform: a modeling study. J. Neurophysiol. 95, 3113–3128. 10.1152/jn.00979.2005 16467426

[B6] Hines M. L. Carnevale N. T. (1997). The neuron simulation environment. Neural Comput. 9, 1179–1209. 10.1162/neco.1997.9.6.1179 9248061

[B7] Hodgkin A. L. Huxley A. F. (1952). Propagation of electrical signals along giant nerve fibres. Proc. R. Soc. Lond. B 140, 177–183. 10.1098/rspb.1952.0054 13003922

[B8] Holt G. R. (1997). A Critical Reexamination of Some Assumptions and Implications of Cable Theory in Neurobiology. Ph.D. Thesis, California Institute of Technology.

[B9] Holt G. R. Koch C. (1999). Electrical interactions via the extracellular potential near cell bodies. J. Comput. Neurosci. 6, 169–184. 10.1023/A:1008832702585 10333161

[B10] Lindén H. Tetzlaff T. Potjans T. C. Pettersen K. H. Grün S. Diesmann M.. (2011). Modeling the spatial reach of the LFP. Neuron 72, 859–872. 10.1016/j.neuron.2011.11.006 22153380

[B11] López-Aguado L. Ibarz J. Herreras O. (2001). Activity-dependent changes of tissue resistivity in the ca1 region *in vivo* are layer-specific: modulation of evoked potentials. Neuroscience 108, 249. 10.1016/S0306-4522(01)00417-1 11734358

[B12] Rall W. (1962). Theory of physiological properties of dendrites. Ann. N. Y. Acad. Sci. 96, 1071–1092. 10.1111/j.1749-6632.1962.tb54120.x 14490041

[B13] Rall W. (1964). Theoretical significance of dendritic trees for neuronal input-output relations, in Neural Theory and Modeling, ed Reiss R. F. (Stanford, CA: Stanford University Press), 73–97.

[B14] Reiter S. (2014). Effiziente Algorithmen und Datenstrukturen für die Realisierung von Adaptiven, Hierarchischen Gittern auf Massiv Parallelen Systemen. Ph.D. dissertation, Universität Frankfurt.

[B15] Scott A. C. (1975). The electrophysics of a nerve fiber. Rev. Mod. Phys. 47, 487–533. 10.1103/RevModPhys.47.487

[B16] Versteeg H. K. Malalasekera W. (2007). An Introduction to Computational Fluid Dynamics: The Finite Volume Method. Harlow: Prentice Hall.

[B17] Vogel A. Reiter S. Rupp M. Nägel A. Wittum G. (2012). Ug 4: A novel flexible software system for simulating pde based models on high performance computers. Comput. Vis. Sci. 16, 165–179. 10.1007/s00791-014-0232-9

[B18] Voßen C. Eberhard J. P. Wittum G. (2007). Modeling and simulation for three-dimensional signal propagation in passive dendrites. Comput. Vis. Sci. 10, 107–121. 10.1007/s00791-006-0036-7

[B19] Xylouris K. Queisser G. Wittum G. (2010). A three-dimensional mathematical model of active signal processing in axons. Comput. Vis. Sci. 13, 409–418. 10.1007/s00791-011-0155-7

